# The interpreter's brain during rest — Hyperconnectivity in the frontal lobe

**DOI:** 10.1371/journal.pone.0202600

**Published:** 2018-08-23

**Authors:** Carina Klein, Silvana Iris Metz, Stefan Elmer, Lutz Jäncke

**Affiliations:** 1 Division of Neuropsychology, Department of Psychology, University of Zurich, Zurich, Switzerland; 2 International Normal Aging and Plasticity Imaging Center (INAPIC), University of Zurich, Zurich, Switzerland; 3 Center for Integrative Human Physiology (ZIHP), University of Zurich, Zurich, Switzerland; 4 University Research Priority Program (URPP), Dynamic of Healthy Aging, University of Zurich, Zurich, Switzerland; 5 Department of Special Education, King Abdulaziz University, Jeddah, Saudi Arabia; Iwate Medical University, JAPAN

## Abstract

Language in its highest complexity is a unique human faculty with simultaneous translation being among the most demanding language task involving both linguistic and executive functions. In this context, bilingually grown up individuals as well as simultaneous interpreters (SIs) represent appropriate groups for studying expertise-related neural adaptations in the human brain. The present study was performed to examine if a domain-specific neural network activation pattern, constituted by brain regions involved in speech processing as well as cognitive control mechanisms can be detected during a task-free resting state condition. To investigate this, electroencephalographic (EEG) data were recorded from 16 SIs and 16 age and gender-matched multilingual control subjects. Graph-theoretical network analyses revealed interhemispheric hyperconnectivity between the ventral part of the prefrontal cortex (pars opercularis and pars triangularis) and the dorsolateral prefrontal cortex (DLPFC) in language experts compared to multilingual controls in the alpha frequency range. This finding suggests that the high cognitive demands placed on simultaneous interpreting lead to an increased neural communication between prefrontal brain regions essentially engaged in supporting executive control—a neural fingerprint that is even detectable during rest.

## Introduction

Simultaneous interpreting is one of the most complex language tasks involving various linguistic and cognitive functions. In fact, it requires a continuous encoding of the input language, the maintenance of the heard information in verbal short-term memory while at the same time translating the information into the output language, inhibiting the articulation of the source, and producing the target language [1, 2]. In the last two decades, first magnetic resonance imaging (MRI) and positron emission tomography (PET) studies have addressed long-term training effects of simultaneous interpreting on the structural [3–5] and the functional [6, 7] architecture of the human brain. Interestingly, results of structural MRI studies revealed differences in brain regions which are also known to be altered in bilinguals. For example, reduced gray matter volume was observed in the middle anterior cingulate gyrus, a brain region which is involved in attentional functions supporting conflict monitoring and error detection [8–10]. Furthermore, gray matter volume was altered in the pars triangularis and its right hemispheric homologue, brain regions playing a role in syntactic processing [11], prosody perception [12], language switching, attention and working memory [13–15]. Also, the left supramarginal gyrus contributing to phonetic processing [16, 17] as well as the brain regions regulating speech production and articulation, i.e. left middle anterior insula [18, 19], caudate nucleus [20] and left pars opercularis [3, 11, 20] were found to have reduced morphological measures. Decreased fractional anisotropy values were found in brain areas regulating speech articulation and sensory-to-motor coupling mechanisms (left anterior insula, upper part of the corticospinal tract, and dorsal part of the right caudate nucleus) as well as controlling interhemispheric information transfer (genu and splenium of the corpus callosum) [4]. A longitudinal MRI study in which subjects were scanned before and after a 15-month period of simultaneous interpreting training showed an increase of cortical thickness over time in brain regions involved in phonetic processing (left posterior superior temporal gyrus, anterior supramarginal gyrus and planum temporale), propositional speech (right angular gyrus), working memory (right dorsal premotor cortex) as well as in executive control functions and attention (right parietal lobule) [5].

In a functional MRI study by Hervais-Adelman and colleagues [7], neural activity during simultaneous interpreting was examined pre- and post-training in contrast to speech repetition (shadowing). The authors found a reduced training-related recruitment in the caudate nucleus, interpreting their findings as a decreased demand on multilingual language control as the task becomes more automatized over the course of an intense training. In 2000, Rinne and colleagues measured brain activation in SIs in a PET scanner during different directions of interpreting, i.e. interpreting into the native language (from L2 to L1), into the non-native language (from L1 to L2—the cognitive more demanding condition) and during shadowing (simultaneous aloud repetition of heard text) [6]. The following contrasts were built: 1) L1 to L2 translation versus shadowing L1, 2) L2 to L1 translation versus shadowing L2 as well as 3) L1 to L2 translation minus shadowing L1 versus L2 to L1 translation minus shadowing L2. Across all contrasts, the authors found mainly left-lateralized effects of simultaneous interpreting: the first condition elicited neural activity in the left frontal lobe (Brodmann area (BA) 6 and BA46). The second condition showed involvement of the left frontal lobe (BA6, BA45), the left inferior temporal zone (BA20/28) as well as the right cerebellum. Contrasting the two interpreting directions (third condition) revealed an increased activation in BA44. The authors showed that cerebral activation patterns vary according to the interpreting direction, recruiting more regions when interpreting in the more effortful direction. Thereby, brain regions are activated that are involved in verbal encoding, processing semantic information, working memory, and when solving effortful tasks.

To the best of our knowledge, only one fMRI study exists that examined spontaneous neural activity during a task-free resting state condition in different types of interpreters: SIs, consecutive interpreters and translators [21]. The authors of this study found hyperconnectivity in SIs compared to the other groups between the left frontal pole and the left middle temporal gyrus as well as between the former region and the left pars opercularis and triangularis.

Despite previous insight into differential brain activation during simultaneous interpreting, to date nothing is known about neural activation patterns during resting state in SIs compared to a multilingual control group. In the context of long-term training and its influence on neural oscillatory activity during resting state, it has repeatedly been shown that repetitive task-specific activations lead to altered network patterns during rest across a variety of domains. For example, a resting state EEG study in professional string players reported increased intra- and interhemispheric functional connectivity between brain regions that are typically involved in music processing and production, such as somatosensory, auditory, and prefrontal regions as well as Broca's area [22]. Expertise-related findings have also been reported in professional chess [23] or badminton players [24] and dancers [25]. These studies emphasize the recruitment of expertise-characteristic brain regions during a resting state period even when not being confronted with the respective stimuli or situation at the moment of data collection.

Thus, instead of examining expertise-specific activation patterns during a task condition, the aim of the present study was to find a putative modulation of resting state network characteristics as a function of long-term simultaneous interpreting training. In this context, we expect to find altered functional connectivity measures in SIs compared to healthy age- and gender matched multilingual control subjects between brain regions that are involved in language processing and control as well as in administrating cognitive control functions, such as Broca's area [3, 11], regions in the ventral and dorsal prefrontal cortex and the posterior part of the middle temporal gyrus and the temporal pole as well as the inferior parietal lobe [5, 6]. Examining EEG-based connectivity measures in the source space allows us to draw conclusions on the underlying brain structures constituting an altered functional network architecture in the SIs compared to the control group. The use of EEG in contrast to fMRI further bears the advantage to disentangle network characteristics in separate frequency ranges. This provides further information on a functionally relevant level than simply discussing the involved brain regions per se. Here, we particularly focused on two frequency ranges being involved in executive control functions such as working memory (i.e. the theta frequency band) [26], inhibition and attention (i.e. the alpha frequency band) [27–29].

## Materials and methods

### Subjects

Sixteen female right-handed SIs, all graduates/students from a local Master's in Applied Linguistics program on simultaneous interpreting (mean age = 34.7, standard deviation (SD) = 9.4 years; age of acquisition of the second language (AoA L2) = 9.4, SD = 3.4; mean age of commencement (AoC) = 27.0, SD = 3.5 years; mean years of experience (YoE) = 7.7, SD = 8.3 years; estimated number of cumulated training hours during life = 4298.1 hours, SD = 6038.9 hours) and sixteen multilingual control subjects (mean age = 34.3, SD = 9.0 years; AoA L2 = 9.9, SD = 3.8) participated in this study. Subjects were matched for handedness [30], age, gender and multilingualism (for detailed information see S1 and S2 Tables). One subject per group grew up bilingually. Language experience was measured with an in-house questionnaire collecting information about mother tongue, bilingualism, foreign languages and the respective AoCs, information about simultaneous interpreting activity, usage of languages (speaking, movie watching, reading, working environment, et cetera) as well as self-reported language proficiency in speaking and writing in everyday communications on a scale from 1 (basic) to 5 (fluent; see S1 File). This questionnaire was used in previous studies [3, 31, 32]. Participants with professional music education were excluded from the study. Musical aptitude was measured with the Advanced Measures of Music Audition (AMMA) test [33]. In this test, participants listen to 30 trials consisting of two successive short piano melodies each. After every single trial, the subject has to decide if the two melodies are either identical or differ in rhythmic or tonal specification from each other. Furthermore, an in-house questionnaire on musical history was applied. In this questionnaire, which was also used in previous studies (see for example [31, 34, 35]), the played musical instruments, musical education as well as training hours per day and week in specific age ranges are indicated (see S2 File). None of the participants reported any current or past neurological, psychiatric, or neuropsychological disease, nor medication or drug abuse. Written informed consent was obtained from all participants and they were paid for participation. The study was approved by the local ethics committee (Kantonale Ethikkommission of the canton of Zurich) according to the declaration of Helsinki.

### Cognitive capability

For testing cognitive capability, participants underwent the KAI (Kurztest für allgemeine Basisgrösse der Informationsverarbeitung) [36] examining the actual cognitive capability (fluid intelligence) based on short-term memory capacity and speed of information processing as well as the MWT-B (Mehrfachwahl-Wortschatz Intelligenztest) [37] testing for crystalline intelligence. In the KAI test, the time for reading aloud four random sequences of visually presented letters as well as the capacity of how many items (numbers and letters, one up to nine items) can be remembered and orally repeated is measured. In the MWT-B, subjects have to choose 37 times the "real" word out of six pseudowords.

### Experimental procedure

Before the EEG recording, participants were tested for their cognitive capabilities and filled out the handedness questionnaire as well as in-house questionnaires about health, music and language skill. After electrode application, participants were placed in a light-dimmed and sound shielded Faraday cage. They sat down in a comfortable chair at a table with a 19-inch monitor at a distance of approximately 75 cm. Subjects were asked to sit calm and relaxed and to avoid strong movements during recording. The EEG paradigm consisted of a task-free period of 3 min eyes open (black fixation cross on a white screen) and 3 min eyes closed (black screen). Afterwards, a verbal Sternberg paradigm was presented according to a previous study by Klein et al. 2016 [38] as well as a post-task resting state period. The present study only covers data analysis of the pre-task resting state condition (3 min eyes open and 3 min eyes closed separately). Presentation of the stimulus material was controlled with the Presentation software (Neurobehavioral Systems, Berkeley, USA, http://www.neurobs.com).

### EEG recording and processing

Continuous EEG was recorded with a 32-channel cap (EASYCAP GmbH, Herrsching, Germany) with a sampling rate of 500 Hz and a high-pass butterworth filter with zero phase distribution (0.1 Hz) by using the BrainVision Recorder Software (Brainproducts, Gilching, Germany, http://www.brainproducts.com). The electrodes (silver/silver chloride sintered ring electrodes) were located at frontal, temporal, parietal and occipital scalp sites according to the international 10–20 system (Fp1, Fp2, F7, F3, Fz, F4, F8, FT7, FC3, FCz, FC4, FT8, T7, C3, Cz, C4, T8, TP9, TP7, CP3, CPz, CP4, TP8, TP10, P7, P3, Pz, P4, P8, O1, Oz, O2). The nose electrode was used as online reference. Electrode impedances were kept below 10 kΩ by applying abrasive electrically conductive gel.

Preprocessing of the data was performed offline by using the BrainVision Analyzer software (version 2.1, Brainproducts, Gilching, Germany, http://www.brainproducts.com). Data were band pass filtered between 0.1–100 Hz (high- and low-pass butterworth filter with zero phase distribution), including a notch filter (50 Hz). An independent component analysis was applied to remove eye blinks and eye movement artifacts [39]. Remaining artifacts were removed by a semi-automatic raw data inspection (maximal allowed voltage steps: 50 μV, maximal allowed differences of values: 200 μV over 200 ms, allowed amplitude range: -200 to 200 μV, lowest allowed activity in intervals: 0.5 μV over 100 ms) and bad channels were interpolated. After artifact cleaning, data were again band pass filtered between 1 Hz and 40 Hz (high- and low-pass butterworth filter with zero phase distribution). The 3 min eyes open and eyes closed resting state periods were segmented into 2 s segments each. Only artifact free segments entered connectivity analysis (a minimum of 74 two second segments per subject; mean number of segments: 88, SD = 2.8).

#### Connectivity analysis in the source space

Functional connectivity is a measure displaying statistical dependencies of neural activity between different regions of interest (ROIs) [40]. In graph-theoretical approaches, a node represents the ROI, while an edge refers to any kind of connection value between the ROIs. As suggested by literature, connectivity analysis in the source space represents a methodological advantage over measuring coherences on the scalp level (between electrodes) and provides simplified result interpretation [41, 42]. Although there is some argument about the reliability of source reconstruction of EEG data, literature exists across diverse conditions [43–45] besides cross-validation studies with functional MRI (fMRI) [46, 47] or PET [48] that show meaningful results for the source reconstruction method used in the present study [49]. Connectivity analyses were performed using the Low Resolution Electromagnetic Tomography (LORETA, Standardized & Exact) KEY toolbox (version 2016–05; http://www.uzh.ch/keyinst/loreta.htm) between all 84 BAs (42 in each hemisphere) implemented in the LORETA KEY software [49–51]. In LORETA, electrically active neuronal generators are calculated based on the recorded electric scalp potentials, which are transformed into current density values (A/cm^2^) for every voxel, assuming similar activation among neighbored voxels. Computations are made in a realistic gray-matter head model using the 152MNI (Montreal Neurological Institute) template by taking into account the effect of volume conduction. In LORETA, the intracerebral space is partitioned into 6239 voxels with a spatial resolution of 5 mm. Because of the assumption of smoothness of data (i.e. neighbored voxels show highly correlated activity), connectivity (here linear instantaneous dependency values, a measure of zero-phase, zero-lag covariances) was calculated between the centroid voxels of the 84 BAs [52]. Linear instantaneous dependencies are a validated measure for calculating distributed networks between predefined brain regions and were repeatedly shown to be affected by long- and short-term training, i.e. in the frame of expertise research [22, 53–55]. The ROI-based transformation matrix was created using the automatic approach. The resulting transformation matrix with a signal-to-noise ratio of 1 was used to calculate instantaneous connectivity values in the theta (4–7 Hz), lower (8–10 Hz) and upper alpha (10.5 Hz—12 Hz) frequency ranges.

As a result, LORETA provides connectivity matrices with linear instantaneous coherence values, one value for every single subject in every single frequency range between all BAs across the 3 min resting state periods. For better result interpretation, the Harvard-Oxford and the Juelich-Histological cortical atlases (http://fsl.fmrib.ox.ac.uk/fsl/fslwiki/Atlases) implemented in the FSL software (http://fsl.fmrib.ox.ac.uk/fsl/fslwiki/FSL) were used for a detailed assignment of the MNI coordinates of the centroid voxels of the ROIs (BAs) to the hyper-connected brain regions, respectively.

### Network-based statistic

For evaluating between-group differences in functional connectivity, the individual connectivity matrices obtained from the LORETA KEY software were subjected to network-based statistic (NBS), separately for the frequency bands of interest [56]. In traditional network analyses, t-tests for each connection of the connectivity matrix are performed and corrected for multiple comparisons without considering dependencies between the connections building a (sub)network. In contrast, NBS tests the network as a whole and is based on a non-parametric supra-threshold cluster test as often applied in fMRI analyses [57]. On the basis of a general linear model approach, the t-test module in NBS was used to compare the individual connectivity matrices between the two groups. By using the component extent option, the extent of functional connections comprising the contrast or effect of interest (i.e., the group difference of the functional connectome during resting state) is examined. For finding a subnetwork in which the two groups differ, sensitivity (set) thresholds are chosen in advance (here t-thresholds) to define which edges form a supra-threshold network. The t-threshold must be chosen arbitrarily in an explorative way. However, as these t-thresholds do not represent the actual alpha error probabilities, this procedure does not affect the false-positive rate of the actual permutation testing. The size, that is the number of edges of the biggest supra-threshold subnetwork, also called the biggest component is then used for test statistics. We controlled the alpha error probability (p < 0.05) by permuting 10000 times the group labels of the subjects and the size of the biggest component. A family-wise error (FWE)-corrected p-value is estimated by counting the number of permutations for which the size of the largest random supra-threshold network is bigger than the one of the real data and dividing it by the number of performed permutations [56]. Here, the connectivity matrices were tested for a range of set thresholds between t = 2.0 to 4.0 in increments of 0.1. Here, we report the biggest obtained subnetworks for t = 3.3. P-values for the network-based measures are one-tailed since a directed contrast is tested. The obtained subnetworks were visualized with the BrainNet Viewer (version 1.53; http://www.nitrc.org/projects/bnv/) [58].

### Statistics of behavioral data and correlation analyses

Demographical data and cognitive capability values were analyzed in the IBM software SPSS Statistics 25 (SPSS Inc, Chicago, IL, USA; https://www.ibm.com/products/spss-statistics). To investigate group comparisons of age and cognitive capability, age and the KAI score were compared with the Mann-Whitney U test (according to deviations from a normal distribution) and a two-tailed unpaired t-test was performed comparing MWT-B values.

To test for brain-behavior relationships, we calculated correlation analyses according to Spearman's rho (two-tailed) between the mean connectivity value of the entire network and the language characteristics YoE, hours of training per week and life in simultaneous interpreting, and AoC.

## Results

### Autobiographical and behavioral data

The two groups did not differ in age (t_(30)_ = 0.115, p = 0.909, two-tailed), AoA L2 (z = -0.710, p = .478) or in performance in the KAI test (z = -1.543, p = 0.123, two-tailed). However, SIs reached a significant higher score in the MWT-B test (t_(30)_ = 2.66, p = 0.012, two-tailed). This might be explained by the fact that the MWT-B relies very much on language experience. Although the control subjects were matched in the number of learned languages, a professional SI is still much more experienced and thus outperforms untrained controls.

### Network-based statistic

Table 1 contains the assignments of the centroid voxels of the BAs constituting the significant network obtained from the NBS analysis to MNI coordinates.

**Table 1:** Specification of Brodmann areas.

BA	MNI coordinates (x, y, z)	Brain region
BA9	(-30, 30, 35)	middle frontal gyrus (dorsolateral prefrontal cortex)
BA9	(30, 30, 35)	middle frontal gyrus (dorsolateral prefrontal cortex)
BA44	(-50, 10, 15)	Broca's area (pars opercularis)
BA44	(55, 10, 15)	right homologue of Broca's area (pars opercularis)
BA45	(-50, 20, 15)	Broca's area (pars triangularis)
BA45	(50, 20, 15)	right homologue of Broca's area (pars triangularis)
BA46	(-45, 35, 20)	middle frontal gyrus (dorsolateral prefrontal cortex)
BA46	(45, 35, 20)	middle frontal gyrus (dorsolateral prefrontal cortex)

MNI coordinates of the centroid voxels representative for the BAs and the respective underlying brain regions constituting the significant network obtained from the NBS analyses.

**Fig 1:**
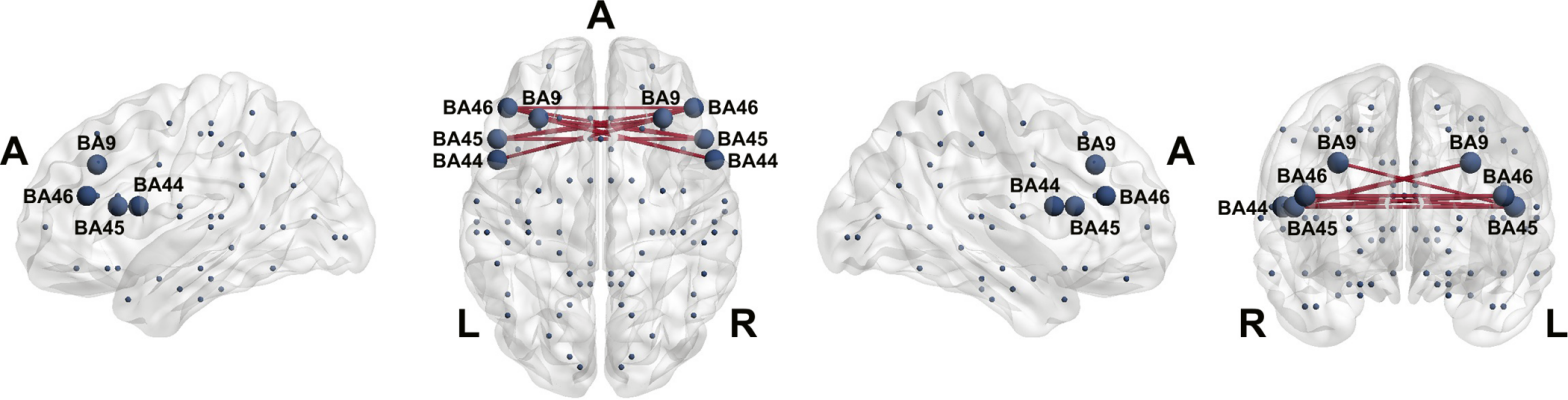
Significant network of the NBS analysis between the SIs and the controls in the lower alpha (8 Hz– 10 Hz) frequency band is shown in the sagittal, horizontal and coronal views (the blue spheres represent the location of the centroid voxels of the 84 BAs that entered the NBS analysis). SIs show increased functional connectivity (depicted in red) in a network containing BA9, BA44, 45 and 46 (enlarged blue spheres) spanning both hemispheres (p = 0.046, FWE-corrected). A = anterior, L = left hemisphere, R = right hemisphere.

The SIs showed increased functional connectivity values only in the lower alpha frequency band for a t-value of 3.3 compared to the control group (SIs: group mean of the connectivity values = 5.42, SD = 0.46; controls: group mean of the connectivity values = 3.78, SD = 0.37; p = 0.046, FWE corrected; effect size r = 0.56 according Cohen's d ≥ 1.35). In particular, the network consisted of eight nodes and eight edges between the left DLPFC, left pars opercularis and pars triangularis and the corresponding homologues in the right hemisphere (see Fig 1). The SIs did not show increased functional connectivity in any other of the tested frequency ranges compared to the controls nor did the controls show significant hyperconnectivities compared to the SIs. Neither did we observe a significant group difference in network connectivity measures during the resting state eyes closed condition.

### Brain-behavior relationships

Correlation analyses between the mean connectivity value of the network obtained from the NBS analysis and autobiographical data of language characteristics within the SIs did not reveal significant results (mean connectivity value with YoE: r = -0.006, p = 0.98; training hours per week: r = 0.074, p = 0.79; training hours during life: r = 0.202, p = 0.45; AoC: r = 0.430, p = 0.10).

## Discussion

In the present source-based EEG study, we revealed an increased functional connectivity matrix in SIs compared to multilingual control participants in the lower alpha frequency band during the eyes open period of the resting state condition. The obtained network was restricted to ventral and dorsal frontal regions across both hemispheres. In particular, SIs demonstrated increased bilateral functional connectivity between the DLPFC and the ventral part of the prefrontal cortex, including pars opercularis, pars triangularis, and their right-sided homologues.

The frontal lobe regulates higher-order cognitive control mechanisms allowing successful performance of complex behavior as well as adapting to environmental changes [59]. Executive functions, an umbrella term for prefrontal processes, include among others attention, working memory, inhibition, initiation, self-monitoring and regulation [60]. Simultaneous interpreting as well as the control of multiple languages as in subjects who grew up bilingually brings along an intense training of higher-order cognitive functions to manage the selection of the proper language, switching between languages or translating and interpreting [3, 9, 61]. In fact, studies in the context of bilingualisms showed that bilingually grown up subjects outperform monolinguals in a variety of linguistic and non-linguistic tasks relying on executive functions such as inhibition [62, 63], working memory [64], attention [62, 65], and set shifting [66].

The sparse literature on neural activation patterns during the process of simultaneous interpreting reports a frontal activation increase in Broca's area when translating into the native language (L2 to L1) and an increase of activation in the left DLPFC when translating into the non-native language [6]. Comparably, literature of bilinguals also report an increased activity in the DLPFC when translating single words compared to word repetition [67]. In particular, an enhanced involvement of the DLPFC [68–70] as well as Broca's area [14] was observed during the process of language switching, and thus, constituting at least a subpart of an attentional network needed for language retrieval. However, to date it is still under debate which executive functions underlie language control. Both the DLPFC as well as Broca's area are discussed in terms of language-specific control processes and in being involved in general language-unrelated cognitive functions [71–74]. For example, described as subserving various functions [13, 75, 76], the Broca's area has gathered considerable attention in the domain of speech and language processing [77–82]. Based on a cytoarchitectonical view, Broca's area can be subdivided in a caudal (pars opercularis, BA 44) and a rostral (pars triangularis, BA 45) part [83]. From a functional perspective, Broca’s area has previously been proposed to contribute to the unification of semantic and syntactic information at the sentence level. In fact, the pars opercularis contributes to planning of articulation, word retrieval memory, syntactic, and phonological processing [20, 78, 81]. Accordingly, pars triangularis has repeatedly been observed during diverse cognitive tasks such as language-related switching mechanisms, attention and inhibition [13–15, 84–86] as well as verbal working memory on both single word [87] and sentence processing [87–89]. Furthermore, an involvement of the pars triangularis has been particularly responsive to syntactic and semantic processes [11, 78, 79, 90–92].

Otherwise, the DLPFC plays an important role in prosodic processing [12, 76, 93] and fundamentally contributes to executive functions [94]. Furthermore, common prefrontal activation patterns in the DLPFC have previously been described during working memory processes and episodic memory processes [95–97]. Evidence from a microstructural point of view argues for a generous amount of inhibitory interneurons in the prefrontal cortex [98], thereby providing an ideal neural basis for cognitive control of language selection by actively suppressing the unwanted output in a face of competition [61]. Thus, the neural underpinnings of bilingualism emphasize the specific role of the prefrontal cortex for mediating inhibition-driven control of language switching, i.e., choosing the proper output language and eventual error correction [15, 70].

Based on 1) such nesting relations between linguistic and cognitive functions in the prefrontal cortex, 2) previous neuroimaging studies indicating structural differences between professional SIs and multilingual control participants in Broca’s area and in its right-sided homologue that correlated with the amount of training [3], and 3) consistent activation patterns in Broca’s area during simultaneous language translation across participants [99], we propose that simultaneous interpreting training may facilitate the neural communication between spatially close areas situated in the ventral and dorsal parts of the prefrontal cortex. Such a processing mode seems to be particularly cost-effective in that it facilitates the local intertwining between linguistic and cognitive functions within local prefrontal circuits. This perspective is not only in line with hierarchical models of speech and language processing [11, 73, 92] but also with a previous diffusion tensor imaging (DTI) study that revealed anatomical differences in the anterior part of the corpus callosum between professional SIs and multilingual control participants [4]. Furthermore, the clustering of prefrontal areas into a densely interconnected functional matrix has previously been shown to be related to enhanced memory performance and cognitive flexibility [100]. This leads to suggest that dynamic training-related functional network reconfigurations constitute a fundamental neural principle underlying executive functions and cognition.

Increased alpha power has consistently been reported to be related to enhanced internally directed attention [101, 102] and to be negatively correlated to neural activity in the dorsal attention network [103]. These findings might lead to suggest that alpha activity is associated with cortical inhibitory processes tuning the brain into an optimized excitatory-inhibitory processing mode by suppressing the activity in neural networks that might interfere with proper processing of an ongoing task [28, 104]. In the context of language processing, mechanisms of language control or switching were described via actively blocking the non-target language by not accessing the mental lexicon of the irrelevant one [15, 105]. Disentangling the functional role of the alpha sub-bands, modulations of lower alpha power were shown to be driven as a function of tonic attentional demands [28, 106] and to be linked to better performance in a working memory task, whereas the upper alpha band seems to be related to stimulus-related cognitive processes [107].

Although we are aware that simultaneous interpreting requires various aspects of cognitive control mechanisms, two facts might support a slight dominance of inhibitory processes in SIs–at least the ones which are detectable during resting state. First, although oscillatory activity in the alpha frequency range is discussed within the frame of attention or working memory, it is also observed to function as a sub-process supporting memory and attention in a phase-dependent inhibitory manner [29]. Second, if working memory would play an important role in SIs we would have also expected to find an altered functional connectivity in the theta frequency range, *the* frequency band of many neural models of working memory, which was however not the case in our study [107, 108]. A more precise interpretation of our results is not possible at the moment lacking cognitive and behavioral data to infer clearly defined brain-behavior relations.

The differences of our findings compared to the ones of the fMRI resting state study by Becker and colleagues on SIs, consecutive interpreters and translators might be explained by the fact that acquisition modalities as well as MNI coordinates of the ROIs differed in the two studies [21]. Furthermore, Becker and colleagues did not have a multilingual control group without any interpreting experience, which makes it difficult comparing both studies directly.

We did not find significant correlations between the mean functional connectivity of our obtained network and language characteristics within the SI group. This does however not necessarily disqualify our findings as being expertise-related. It rather leads us to assume that either the variable mediating functional connectivity in SIs has not been properly acquired or that the obtained network displays a domain-specific predisposition rather than an experience- or training-related neural activity pattern. Therefore, future studies should for example apply approved objective language tests instead of self-assessed proficiency levels in a longitudinal approach.

The fact that the two groups did only differ in connectivity measures during the eyes open condition is not surprising. Previous research has shown that oscillatory activity during rest with eyes open is not directly comparable with neural activation patterns during an eyes closed resting state period. Specifically, the dominant alpha oscillations observable during the eyes closed period are desynchronized or blocked when opening the eyes [109]. Besides this alpha dominance mediated by the reticular activation system [110, 111], alpha power also shows a positive relation with the arousal level, i.e. the current energetic level of the organisms [112] of the recorded subject across different resting state conditions. In this context, Barry and colleagues showed that the eyes open period was associated with an increased arousal level and a global reduction in alpha power compared to the eyes closed condition [113]. This conditional effect of alpha frequency power across the two resting state conditions recorded in the present study might have led to the fact that the expertise-related resting state network obtained during the eyes open period in the oscillatory alpha range was superimposed by the physiologically-driven change in alpha power during the eyes closed period. Differences across these two conditions have also been reported with regard to connectivity measures and in a graph-theoretical framework in both the alpha and theta frequency bands [114, 115]. In addition, as SIs interpret with eyes open, it is not surprising that a training-related network alteration appears in the more comparable condition.

To summarize, as being the first EEG study on the EEG source level, our results make a further contribution to the influence of language expertise on the intrinsic imprint in network activity patterns during a task-free resting state condition. Comparable to previous resting state studies in the context of expertise research, we found an altered connectivity in networks constituted of typical domain-related nodes. Our results support the idea of a training-related functional network configuration in SIs facilitating challenging language tasks by intertwining general cognitive and language-related processing. However, since we examined resting state network activity, the interpretation still remains speculative and therefore, further research in this area is needed.

## Limitations

One limitation is that only female subjects participated in the present study. This is owed to the fact that it was nearly impossible to find enough male SIs. Besides diverse differences in resting state electroencephalographic activity and brain anatomy [116] between male and females [117], sexual dimorphisms of brain regions involved in speech processing have been observed [118–121]. In particular, language processing in females seems to be more bilaterally represented than in males who show a stronger left-dominant lateralization [122, 123]. Thus, future studies comparing network characteristics during a task-free condition between male and female (controlled for menstrual phase) SIs are necessary to draw straightforward conclusions. Furthermore, including a control group of other language experts, such as consecutive interpreters or translators might help to further unravel cognitive control mechanisms in SIs and the influence of language proficiency on connectivity patterns.

## Supporting information

S1 File English and German version of the in-house questionnaire on language skills. (PDF)

S2 File English and German version of the in-house questionnaire on musicality. (PDF)

S3 File Minimal data set. (XLSX)

S1 Table Autobiography of language characteristics. The age of acquisition (AoA), the years of experience (YoE), and self-reported linguistic skills (LS) in speaking (s) and writing (w) are listed in parenthesis for every learned language. Mother tongue is printed in bold. SI = simultaneous interpreter, C = control subject.
(PDF)

S2 Table Interpreting directions of the simultaneous interpreters. The symbol ‘↔’ means that SIs can translate into both languages, whereas ‘→’ means that SIs can only work in one direction. SI = simultaneous interpreter.
(PDF)
