# Supplementary material for: The interpreter's brain during rest — Hyperconnectivity in the frontal lobe
Source: PLoS One. 2018 Aug 23;13(8):e0202600. doi: 10.1371/journal.pone.0202600 (PMC6107212; doi:10.1371/journal.pone.0202600)
Supplement: S1 File — (PDF) [file pone.0202600.s001.pdf]

University of Zurich[UZH]

Division of Neuropsychology
Department of Psychology

Questionnaire on language skills

Subject code: ....
Age: ....

Did you grow up bilingually? Yes □ No □
…with two languages in the family? Yes □ No □
…with different languages in family and school? Yes □ No □

At what age did you acquire the second language?
....

What is/are your mother tongue(s):
....

What foreign languages do you speak? Please indicate in descending order with regards to proficiency.
....
....
....
....

At what age did you start learning the respective foreign languages? Since when/for how many years have you spoken the languages?
....
....
....
....

Do you read books? Yes □ No □

In which languages do you read books?
....

How often and for how long do you read books for your study/work or free-time on average per week (hours per week)?

study/work: ....

free-time: ....

If you had the choice, which language would you choose for reading a book if you do not master the original language of the book?

....

Please list under which circumstances you are/were confronted with the respective languages:

television: ....

listening to music: ....

reading: ....

interaction with the family: ....

interaction with friends: ....

study/work: ....

Do you interpret simultaneously? Yes ☐ No ☐

If yes, please indicate in which direction: ↔ →

....

....

....

....

Since when have you mastered simultaneous interpreting?

....

How many hours per week do you interpret simultaneously?

....

Please indicate in the following on a 5-point Likert scale how proficient you are in each of the languages.

1 = limited knowledge (very easy conversations, e.g. ordering drinks in a restaurant)
2 = average knowledge (easy conversations, e.g. route descriptions)
3 = good knowledge (able to hold a conversation but limited knowledge of colloquial phrases)
4 = very good knowledge (spontaneous speaking is no problem)
5 = excellent knowledge (diverse situations can be mastered perfectly, e.g. emotions can be phrased easily as well as the use of colloquial phrases and jokes)

Language		1	2	3	4	5
	oral					
	written					
	oral					
	written					
	oral					
	written					
	oral					
	written					
	oral					
	written					

University of Zurich[UZH]

Psychologisches Institut
Lehrstuhl für Neuropsychologie

Evaluationsformular Sprachkenntnisse

Probandencode: ....

Alter: ....

Sind Sie bilingual aufgewachsen? Ja ☐ Nein ☐

…mit zwei Sprachen innerhalb der Familie? Ja ☐ Nein ☐

…mit unterschiedlichen Sprachen in der Familie/Schule? Ja ☐ Nein ☐

In welchem Alter wurde die zweite Sprache erlernt?

....

Bitte geben Sie Ihre Muttersprache(n) an:

....

Welche Fremdsprachen sprechen Sie? Bitte in absteigender Reihenfolge geordnet nach Professionalität aufführen.

....

....

....

....

In welchem Alter haben Sie die jeweilige Fremdsprache erlernt? Seit wieviel Jahren sprechen Sie die jeweiligen Sprachen bzw. über wieviel Jahre wurden sie gesprochen?

....

....

....

....

Lesen Sie Bücher? Ja ☐ Nein ☐

In welchen Sprachen lesen Sie Bücher?

....

Wie oft und wie lange lesen Sie im Durchschnitt Bücher fürs Studium/Beruf und in ihrer Freizeit (Stunden pro Woche)?

Studium/Beruf: ....
Freizeit: ....

Wenn Sie wählen könnten, in welcher Sprache würden Sie ein Buch lesen (davon ausgehend, dass Sie die Originalsprache des Buches nicht beherrschen)?
....

Bitte führen Sie auf, unter welchen Umständen Sie mit den verschiedenen Sprachen konfrontiert werden/wurden:
Fernsehen: ....
Musik hören: ....
Lesen: ....
Interaktion mit der Familie: ....
Interaktion mit Freunden: ....
Studium/Beruf: ....

Können Sie simultan dolmetschen? Ja ☐ Nein ☐

Wenn ja, geben Sie bitte die Richtung der Sprachen an: ↔ →

....
....
....
....

Seit wann beherrschen Sie das simultane Dolmetschen?
....

Wie viele Stunden pro Woche dolmetschen Sie simultan?
....

Bitte geben Sie im Folgenden auf einer 5-stufigen Skala an, wie gut Sie die jeweiligen Sprachen beherrschen.

1 = geringe Kenntnisse (sehr einfache Unterhaltungen sind möglich, z.B. Getränkebestellung im Restaurant)
2 = mittlere Kenntnisse (einfache Gesprächssituation sind möglich, z.B. Wegbeschreibungen)
3 = gute Kenntnisse (Gesprächssituationen können bewältigt werden, aber Redewendungen im Alltag und spontanes Sprechen gelingen nur mässig)
4 = sehr gute Kenntnisse (spontanes Sprechen stellt kein Problem dar)
5 = exzellente Kenntnisse (es kann in unterschiedlichen Situationen angemessen kommuniziert werden. Gefühle können ohne Einschränkung ausgedrückt werden und auch eine humorvolle Anwendung der Sprache gelingt (Witze, etc.))

**Sprache**		**1**	**2**	**3**	**4**	**5**
	mündlich					
	schriftlich					
	mündlich					
	schriftlich					
	mündlich					
	schriftlich					
	mündlich					
	schriftlich					
	mündlich					
	schriftlich					
