# Supplementary material for: The interpreter's brain during rest — Hyperconnectivity in the frontal lobe
Source: PLoS One. 2018 Aug 23;13(8):e0202600. doi: 10.1371/journal.pone.0202600 (PMC6107212; doi:10.1371/journal.pone.0202600)
Supplement: S2 File — (PDF) [file pone.0202600.s002.pdf]

Questionnaire on musicality

Subject code: ....
Age: ....

Do you have absolute pitch? Yes ☐ No ☐
Do you play a musical instrument? Yes ☐ No ☐

At what age did you start playing a musical instrument?
....

What was the first musical instrument you played?
....

At what age did you start playing additional musical instruments and which ones?
....
....
....
....

What music education did you absolve?
....
....
....

What was the main musical instrument you played?
....

Please indicate how many hours you played the main musical instrument per day and week within the last year:
Average number of training hours per day: ....
Average number of training hours per week: ....

Do you listen to music in your free time? Yes □ No □

How much did you listen to music during the last year on average per day?
....

How do you listen to music (stereo system, headphones, in-ear earphones)?
....

What music genres do you listen to?
....

Evaluation of training intensity

Please indicate at what age you practiced which musical instrument on average per day and per week.

Age (years)	Hours per day	Hours per week	Musical instrument
0-7			
8-10			
11-13			
14-16			
17-19			
20-22			
23-25			
26-28			
29-31			
32-34			
35-37			
38-40			

Evaluationsformular Musikalität

Probandencode: ....

Alter: ....

Haben Sie ein absolutes Gehör? Ja ☐ Nein ☐

Spielen Sie ein Instrument? Ja ☐ Nein ☐

In welchem Alter haben Sie angefangen zu musizieren?

....

Mit welchem Instrument haben Sie angefangen zu musizieren?

....

In welchem Alter haben Sie zusätzliche Instrumente gespielt?

....

....

....

....

Welche Musikausbildung haben Sie absolviert?

....

....

....

Welches ist Ihr Hauptinstrument?

....

Bitte geben Sie an, wie intensiv Sie im vergangenen Jahr Ihr Hauptinstrument trainiert haben:

Durchschnittliche Anzahl Trainigsstunden pro Tag: ....

Durchschnittliche Anzahl Trainigsstunden pro Woche: ....

Hören Sie in ihrer Freizeit Musik? Ja ☐ Nein ☐

Wie oft haben Sie im vergangenen Jahr im Durchschnitt pro Tag Musik gehört?

....

Wie hören Sie Musik (Musikanalage, Kopfhörer, In-ear Kopfhörer)?

....

Welche Musikgenres hören Sie?

....

Evaluation der Trainingsintensität

Bitte geben Sie in der folgenden Tabelle an, in welchem Alter Sie durchschnittlich pro Tag und pro Woche welches Instrument gespielt haben.

Alter (Jahre)	Stunden pro Tag	Stunden pro Woche	Instrument
0-7			
8-10			
11-13			
14-16			
17-19			
20-22			
23-25			
26-28			
29-31			
32-34			
35-37			
38-40			
