# Supplementary material for: The interpreter's brain during rest — Hyperconnectivity in the frontal lobe
Source: PLoS One. 2018 Aug 23;13(8):e0202600. doi: 10.1371/journal.pone.0202600 (PMC6107212; doi:10.1371/journal.pone.0202600)
Supplement: S1 Table — The age of acquisition (AoA), the years of experience (YoE), and self-reported linguistic skills (LS) in speaking (s) and writing (w) are listed in parenthesis for every learned language. Mother tongue is printed in bold. SI = simultaneous interpreter, C = control subject. (PDF) [file pone.0202600.s004.pdf]

S1 Table: Autobiography of language characteristics.

Subject	Age	Learned languages (AoA; YoE; LS in s; LS in w)
SI 1	46	**German** (0, 46, 5, 5), French (11, 35, 4, 4), English (12, 34, 5, 5), Latin (12, NA, NA, NA), Italian (15, NA, 2, 2), Spanish (20, NA, 1, 1)
SI 2	33	**Italian** (0, 33, 5, 5), French (8, 25, 5, 5), German (13, 20, 3, 4), English (20, 13, 4, 5), Spanish (NA, NA, 1, 4), Portuguese (NA, NA, 1, 4)
SI 3	26	**German** (0, 26, 5, 5), French (9, 17, 5, 5), English (11, 15, 5, 5), Spanish (14, 14, 5, 5)
SI 4	23	**German** (0, 23, 5, 5), French (9, 14, 5, 5), English (11, 12, 5, 5), Italian (14, 9, 4, 4), Japanese (14, 3, 1, 1), Chinese (19, 3, 1, 1)
SI 5	26	**German** (0, 26, 5, 5), English (12, 14, 5, 5), French (12, 14, 3, 4), Spanish (14, 12, 4, 4), Portuguese (21, 5, 4, 4)
SI 6	27	**German** (0, 27, 5, 5), French (11, 16, 4, 3), English (13, 14, 5, 5), Italian (16, 1, 1, 1), Russian (18, 3, 1, 1), Czech (19, 8, 1, 1)
SI 7	26	**German, English** (0, 26, 5, 5), French (11, 15, 3, 3), Spanish (16, 10, 4, 4), Italian (NA, NA, 1, 1)
SI 8	52	**German** (0, 52, 5, 5), French (12, 40, 4, 4), Latin (12, 18, NA, NA), English (14, 38, 5, 5), Spanish (16, 36, 4, 4), Italian (27, 25, 4, 4)
SI 9	29	**German** (0, 29, 5, 5), English (10, 19, 5, 5), French (11, 18, 4, 3), Spanish (15, 14, 4, 4), Croatian (24, 5, 4, 4), Italian (NA, NA, 1, 1)
SI 10	40	**Macedonian** (0, 40, 5, 5), Serbian (6, 34, 5, 5), English (10, 30, 5, 5), Spanish (20, 20, 2, 2), Greek (22, 18, 2, 2), Swedish (24, 16, 2, 2), German (34, 6, 3, 3)
SI 11	43	**German** (0, 43, 5, 5), French (13, 30, 5, 5), English (15, 28, 5, 5), Italian (15, 3, 3, 3), Swedish (18, 25, 5, 5), Russian (22, 5, 3, 2)
SI 12	32	**German** (0, 32, 5, 5), English (7, 25, 5, 5), French (11, 21, 4, 4), Spanish (15, 17, 3, 3)
SI 13	32	**Italian** (0, 32, 5, 5), English (14, 18, 5, 5), French (14, 18, 5, 5), German (15, 17, 5, 5), Russian (27, 5, 4, 4), Arabic (31, 1, 1, 1)
SI 14	50	**Italian** (0, 50, 5, 5), French (9, 41, 4, 4), English (12, 38, 5, 5), German (12, 38, 5, 5), Spanish (33, 17, 4, 4)
SI 15	42	**German** (0, 42, 5, 5), English (10, 32, 4, 4), French (20, 22, 5, 5), Italian (21, 3, 3, 3), Thai (NA, NA, 2, 1)
SI 16	28	**German** (0, 28, 5, 5), French (10, 18, 4, 4), English (12, 16, 5, 5), Spanish (15, 13, 4, 4)

C 1	30	**German** (0, 28, 5, 5), French (11, 19, 3, 3), English (15, 15, 4, 4), Spanish (17, 13, 4, 4), Italian (NA, NA, 1, 1), Arabic (NA, NA, 1, 1), Indonesian (NA, NA, 1, 1)
C 2	27	**German** (0, 27, 5, 5), Serbian (12, 15, 4, 4), English (13, 14, 4, 4), French (13, 14, 1, 1), Italian (16, 11, 2, 2)
C 3	46	**German** (0, 46, 5, 5), English (12, 34, 4, 4), French (14, 32, 2, 2), Tibetan (40, 6, 2, 2)
C 4	26	**German, Greek** (0, 26, 5, 5), French (10, 6, 2, 1), English (13, 13, 4, 4)
C 5	40	**Russian** (0, 40, 5, 5), German (11, 29, 5, 5), English (18, 22, 4, 4)
C 6	30	**Luxembourgish** (0, 30, 5, 5), German (7, 23, 5, 5), French (7, 23, 5, 4), English (14, 16, 4, 3)
C 7	24	**Armenian** (0, 24, 5, 5), Russian (12, 12, 3, 5), English (13, 11, 5, 5), Italian (19, 5, 3, 4), German (21, 3, 5, 5)
C 8	29	**Lithuanian** (0, 29, 5, 5), German (10, 7, 4, 4), Russian (12, 17, 2, 2), English (20, 8, 4, 4), French (23, 6, 1, 1)
C 9	53	**German** (0, 53, 5, 5), French (13, 6, 2, 2), English (14, 9, 2, 3), Japanese (52, 0.5, 1, 1)
C 10	25	**English** (0, 25, 5, 5), German (11, 14, 5, 5), Polish (12, 13, 4, 2), French (12, 13, 4, 2), Spanish (12, 13, 4, 3), Portuguese (12, 13, 3, 1)
C 11	30	**German** (0, 30, 5, 5), French (11, 19, 4, 3), Italian (12, 18, 1, 2), English (13, 17, 5, 5), Spanish (15, 15, 5, 5)
C 12	28	**German** (0, 28, 5, 5), Dutch (3, 25, 5, 3), French (10, 18, 2, 2), English (12, 16, 4, 3), Spanish (20, 8, 5, 5), Latin (23, 5, 2, 5), ancient Greek (25, 3, 1, 5), Italian (28, 0.5, 2, 1)
C 13	34	**German** (0, 34, 5, 5), French (8, 26, 5, 4), English (12, 22, 4, 4), Spanish (19, 15, 2, 1), Swedish (34, 0.5, 1, 1)
C 14	36	**Polish** (0, 36, 5, 5), German (11, 7, 5, 5), English (15, 20, 5, 5), Chinese (21, 15, 4, 4), Russian (31, 0.5, 2, 1)
C 15	46	**German** (0, 46, 5, 5), French (16, 30, 3, 3), Italian (17, 29, 3, 3), English (18, 28, 3, 3), Spanish (46, 0.5, 3, 1)
C 16	45	**German** (0, 45, 5, 5), French (11, 44, 4, 3), English (16, 29, 4, 3), Italian (NA, NA, 1, 1)

The age of acquisition (AoA), the years of experience (YoE), and self-reported linguistic skills (LS) in speaking (s) and writing (w) are listed in parenthesis for every learned language. Mother tongue is printed in bold. SI = simultaneous interpreter, C = control subject.
