# Supplementary material for: The interpreter's brain during rest — Hyperconnectivity in the frontal lobe
Source: PLoS One. 2018 Aug 23;13(8):e0202600. doi: 10.1371/journal.pone.0202600 (PMC6107212; doi:10.1371/journal.pone.0202600)
Supplement: S2 Table — The symbol ‘↔’ means that SIs can translate into both languages, whereas ‘→’ means that SIs can only work in one direction. SI = simultaneous interpreter. (PDF) [file pone.0202600.s005.pdf]

S2 Table: Directions of simultaneous interpreting of the SIs.

Subject	Interpreting directions			
SI 1	German ↔ English	French → German		
SI 2	Italian ↔ English	French → Italian	German → Italian	
SI 3	German ↔ English	French → German	Spanish → German	
SI 4	German ↔ English	French → German	Italian → German	
SI 5	English → German	French → German	Spanish → German	Portuguese → German
SI 6	German ↔ English	French → German		
SI 7	German ↔ English	French → German	Spanish → German	
SI 8	English → German	French → German	Spanish → German	Italian → German
SI 9	English → German	French → German	Spanish → German	Croatian → German
SI 10	Macedonian ↔ English	Macedonian ↔ Serbian	German → Macedonian	
SI 11	German ↔ English	French → German	Swedish → German	
SI 12	German ↔ English	French → German	Spanish → German	
SI 13	German → Italian	English → Italian	French → Italian	
SI 14	German → Italian	English → Italian	French → Italian	Spanish → Italian
SI 15	German ↔ French	English → German		
SI 16	German ↔ English	French → German		

The symbol '↔' means that SIs can translate into both languages, whereas '→' means that SIs can only work in one direction.
